# Effects of Electromyographic Biofeedback on Functional Recovery of Patients Two Months after Total Knee Arthroplasty: A Randomized Controlled Trial

**DOI:** 10.3390/jcm11113182

**Published:** 2022-06-02

**Authors:** Iva Sklempe Kokic, Matko Vuksanic, Tomislav Kokic, Ivan Peric, Ivana Duvnjak

**Affiliations:** 1 Faculty of Kinesiology Osijek, Josip Juraj Strossmayer University of Osijek, 31000 Osijek, Croatia; tkokic@gmail.com (T.K.); ivan.peric@kifos.hr (I.P.); ivana.duvnjak@kifos.hr (I.D.); 2 Bizovacke Toplice Rehabilitation Hospital, 31222 Bizovac, Croatia; matko.vuksanic@bizovacke-toplice.hr; 3 Department of Health Studies, College of Applied Sciences “Lavoslav Ruzicka” in Vukovar, 32000 Vukovar, Croatia; 4 Faculty of Medicine, Josip Juraj Strossmayer University of Osijek, 31000 Osijek, Croatia; 5 County General Hospital Vinkovci, 32100 Vinkovci, Croatia; 6 Faculty of Dental Medicine and Health, Josip Juraj Strossmayer University of Osijek, 31000 Osijek, Croatia

**Keywords:** biofeedback, knee replacement, rehabilitation, quality of life, physical functional performance

## Abstract

The incidence of total knee arthroplasty (TKA) is steadily increasing worldwide. Therefore, it is crucial to develop efficient rehabilitation protocols and investigate the innovations in medical technology, which could improve rehabilitation outcomes. The aim of the study was to investigate the effect of adding electromyographic biofeedback (EMG-BF) to the conventional program of rehabilitation after TKA on quality of life, intensity of pain, and functional performance. The study was designed as a randomized controlled trial. A total of 131 patients were randomly assigned to two groups: an experimental group (*n* = 67; median age 70 (IQR 10)), and a control group (*n* = 64; median age 69 (IQR 9)). Both groups participated in an inpatient program of 21 days of rehabilitation, including land-based and aquatic exercise therapy, electrotherapy, and education. In the experimental group, a portion of land-based exercise therapy was supplemented by EMG-BF. A numeric rating scale (NRS), Knee Injury and Osteoarthritis Outcome Score (KOOS), use of mobility aids, 30 s chair stand test (CST), and timed up and go (TUG) test were used to measure outcomes. Both groups improved their functional abilities from day 1 to day 21 of rehabilitation. A higher proportion of participants did not use a walking aid (*p* < 0.002), and their NRS, KOOS, 30 s CST and TUG scores improved (*p* < 0.001). There were no significant differences between the groups in the outcomes. EMG-BF did not provide additional benefits to the conventional rehabilitation after TKA.

## 1. Introduction

Knee osteoarthritis is one of the most frequent musculoskeletal degenerative disorders in older age. The knee joint has an important role in mobility and activities of daily life, and pain and lack of knee function greatly affect the quality of life [1]. In advanced stages of osteoarthritis, patients face progressive disability and lowered quality of life, and the healthcare system faces higher costs [2]. Established conservative treatments for knee osteoarthritis include exercise therapy [3], knee bracing [4], physical modalities [5], and pharmacotherapy [6], but their long-term effectiveness is limited; thus, total knee replacement is ultimately needed for the majority of patients with knee osteoarthritis.

Therefore, many older adults undergo total knee arthroplasty (TKA) to restore their ability to walk, independence, and quality of life. TKA has become one of the most common orthopedic procedures [7], and its incidence has increased steadily since the introduction of the procedure and continues to increase in all age groups with the goal of relieving symptoms of osteoarthritis and restoring function [8].

Efficient rehabilitation is crucial for restoring function following TKA [9]. Patients after TKA often experience pain and impaired muscle strength, especially of m. quadriceps femoris and decreased neuromuscular control [10], which is dealt with during the postoperative rehabilitation. These problems affect patients’ function and satisfaction. Restoring m. quadriceps femoris strength is a common goal following TKA. Most rehabilitation protocols include therapeutic exercise and physical agents such as cryotherapy and electrotherapy [11,12].

Despite significant efforts to improve the outcomes after TKA, many patients following TKA experience persistent muscle weakness, functional impairment, and pain in the long term [13,14,15]. Therefore, investigating the efficiency of innovations in medical technology which could improve rehabilitation outcomes for this group of patients is rather important. Recent innovations in rehabilitation technology have to be verified to prove their clinical meaningfulness and justify their cost. One of the recent innovations in medical rehabilitative technology is electromyographic biofeedback (EMG-BF), which could hypothetically improve m. quadriceps femoris strength and therefore positively impact functional outcomes after TKA and offer additional benefits to these patients. Its usefulness after TKA is still rather unexplored.

Electromyographic biofeedback is an additional treatment modality that can be used to modulate muscle contraction by bringing the muscular tension to the level of consciousness [16]. It is the technique of providing biological information to patients in real-time that otherwise would be unknown [17]. EMG-BF is used in combination with conventional rehabilitation with the goal of improving strength and functionality for various orthopedic conditions. The evidence regarding its efficacy after TKA is still limited, but a recent systematic review showed its potential in controlling pain and improving m. quadriceps femoris strength and functionality after TKA [16].

The objective of this study was to examine the effects of adding EMG-BF to the conventional program of rehabilitation after TKA, consisting of therapeutic exercise and electrotherapy, on objective and subjective functional outcomes, including pain, patient-reported measure of quality of life, lower body strength, mobility, and functional performance. This would add new evidence to the body of scientific knowledge regarding the optimal design of rehabilitation protocols after TKA and improve patients’ outcomes. If EMG-BF is found to have significant effects on patients’ recovery, then its use could be justified in postoperative rehabilitation after TKA. We hypothesized that adding EMG-BF to the conventional rehabilitation program would improve the objective and subjective functional outcomes of patients.

## 2. Materials and Methods

### 2.1. Study Design and Population

The study was carried out as a prospective, parallel-group, single-blinded, randomized controlled trial on patients after TKA attending inpatient postoperative rehabilitation at Bizovacke Toplice Rehabilitation Hospital, Bizovac, Croatia, between November 2018 and December 2019. The study was not blinded to participants because of its nature. However, the assessors were blinded.

Patients were recruited by direct contact after checking into a rehabilitation institution. Inclusion criteria were as follows: (1) patients of both genders after TKA without previous postoperative inpatient postoperative rehabilitation; (2) age between 18 and 79 years; and (3) ability to read, understand, and speak Croatian language.

Exclusion criteria were: (1) patients after revision TKA, (2) patients with comorbidities that did not allow normal mobility due to other causes (e.g., hemiparesis, severe diseases, and conditions affecting internal organs), (3) non-ambulatory patients before TKA where surgery was performed only for pain relief, and (4) patients not able to follow standard institutional rehabilitation protocol.

Ethical approval was obtained from the Ethics Committee of the Bizovacke Toplice Rehabilitation Hospital, Bizovac, Croatia (no. 71/2018/I), and the trial was registered with The Australian New Zealand Clinical Trials Registry (ACTRN12618001782224). Participants gave their written informed consent. The trial was conducted in accordance with the Declaration of Helsinki.

### 2.2. Randomization

When patients satisfied all inclusion criteria and gave their written informed consent, they were randomly assigned into 2 groups: experimental (EG) and control (CG). We used a computerized randomization stratification procedure with the 1:1 ratio in permuted blocks of four to ensure a similar number of participants between groups. The assessors were blinded to the group allocation of participants.

### 2.3. Sample Size and Power Analysis

Before conducting the study, the sample size was calculated using G*Power Software [18], considering timed up and go test (TUG) as one of the primary outcome measures, based on the results of studies published by Yuksel et al. [19] and Mizner et al. [20]. Considering a power of 80%, an effect size of 0.05, a one-sided 0.05 significance level, and a 15% dropout rate, 117 patients would be necessary to detect a 2.27 s difference between the two groups, which is a minimal clinically important difference for this outcome measure.

### 2.4. Rehabilitation Protocol

Both groups participated in an inpatient program, which included 21 days of postoperative rehabilitation after TKA according to standard institution’s protocol, which consists of daily sessions of land-based exercise therapy, application of physical agents (interferential current therapy and electrostimulation), group aquatic exercise, and individual education.

Each of the 21 days of rehabilitation followed the same protocol, except on Sundays when patients did not receive physiotherapy. On average, there were three Sundays during the patients’ stay at the rehabilitation institution, and there were 18 days of rehabilitation consisting of 50 min of land-based exercise, 30 min of aquatic exercise, 10 min of interferential current therapy, and 10 min of electrostimulation. All interventions were performed on a daily basis, except individual education, which was performed once. The intervention was provided once per day, face to face, by a physiotherapist in a special hospital for rehabilitation. Adherence to the treatment was monitored by the physiotherapist in charge.

Program of land-based exercise consisted of 20 exercises. Patients performed 7 variations of isometric exercises for thigh muscles and 13 dynamic exercises for lower limbs, which include active straight leg raise (upward and combined upward and into hip abduction) from a long-sitting position with and without elastic band, hip abduction straight leg raise (and in combination with hip flexion and extension), hip flexion in long-sitting position, leg extension in sitting position with an elastic band, hip abduction from sitting position, hip flexion from sitting position, hip flexion and leg extension exercise on a Swiss ball, and pelvic lift exercise. Isometric exercises were performed in sets of 1, with 5 repetitions with a maximal effort lasting 5 s. Dynamic exercises were performed in 1–2 sets, with 10 repetitions. Progression of the dynamic exercise was achieved with an elastic band, and during the last 6 days of rehabilitation, ankle weights (1 kg) were included.

Interferential current therapy was provided using Myomed 632 device (Enraf-Nonius B. V., Rotterdam, The Netherlands) for a duration of 10 min using symmetrical waves, phase duration of 100 µs, phase interval 0 µs, impulse frequency 80 Hz, modulation frequency 0 Hz, modulation program 1/1 s, with intensity in mA according to patient’s tolerance.

Electrostimulation was provided using Myomed 632 device (Enraf-Nonius B. V., Rotterdam, The Netherlands) for a duration of 10 min using symmetrical waves, phase duration of 500 µs, phase interval 0 µs, impulse frequency 1 Hz, modulation frequency 0 Hz, modulation program 1/1 s, with intensity in mA according to patient’s tolerance.

Group aquatic exercise included 13 exercises for range of motion and strength (standing toe raise, standing heel raise, semi-squats, hip flexion, extension and abduction in standing position and while floating in the water, knee flexion and extension in standing position and while floating in water).

Individual education was mainly targeted to the long-term care of the prosthesis regarding how to return to activities of daily life, recommended and non-recommended activities, coping with postoperative pain, and prevention of blood clots by regular physical activity. It was provided by physiotherapists once, at the start of the rehabilitation, with additional explaining as necessary (if patients had questions) during further rehabilitation sessions.

#### 2.4.1. Experimental Biofeedback-Assisted Exercise Therapy

In the experimental group, a portion of land-based exercise therapy was biofeedback-assisted (Myomed 632, Enraf-Nonius B. V., Rotterdam, The Netherlands), with individually adjusted targets for muscular isometric contraction, according to the manufacturer’s instruction. Biofeedback was provided by superficial electromyography (EMG) unit, e.g., electrical activity of a muscle is registered and passed on as quantitative information (feedback) to the patient and the physiotherapist in real-time, during exercise. That way, the patients were able to monitor the strength of the contraction on screen and try to achieve adequate intensity of the muscular isometric contraction.

The device was attached to the patient via three electrodes, two EMG electrodes, and one reference electrode. The EMG electrodes were placed on the muscle belly of the m. quadriceps femoris according to manufacturer’s instruction. The reference electrode was placed on the anterior portion of the tibia of the opposite leg. At the beginning of the biofeedback-assisted exercise, the patient performed maximal isometric contraction of the m. quadriceps femoris to establish the threshold, e.g., the target intensity of the muscular contraction. The sensitivity of the EMG signal was set to 200 µV to achieve good visibility to the patient, e.g., to achieve a good graphical representation of the muscle’s electrical activity on the screen of the device. After the patient performed maximal isometric contraction, this was recorded by the device.

The physiotherapist in charge set the threshold, which was the value of the muscular contraction the patient should accomplish during the exercise, e.g., during periods of active muscular contraction. The patient should have contracted the muscle above the threshold, which was visible on the screen. The threshold was set to 80% of the maximal isometric contraction achieved at the start of the session. The biofeedback-assisted exercise consisted of isometric contractions of the m. quadriceps femoris for 15 min (10 s periods of contraction and 10 s periods of relaxation between contractions).

#### 2.4.2. Conventional Rehabilitation Protocol

The control group was given the same rehabilitation protocol except for biofeedback-assisted exercise. They performed this portion of isometric exercises without biofeedback assistance.

### 2.5. Outcome Measures

Baseline information, taken at the initial interview on the first day of rehabilitation, included demographic data, medical history, height and body mass, side of the operated knee, level of the constraint of TKA, surgical technique and approach, place of the surgery and whether it was performed at the university hospital or general hospital, postoperative day, and mobility aid currently in use (one crutch, two crutches, walker or no mobility aid used).

Body mass index (BMI) was calculated according to the standard equation. Both groups had their assessments and measurements taken on their 1st and 21st day of inpatient rehabilitation. They included the self-reported functional status of the knee using Knee Injury and Osteoarthritis Outcome Score (KOOS), Croatian version LK1.0, pain intensity measured by numeric rating scale (NRS), functional status of the lower extremity using 30 s chair stand test (CST), and patients’ mobility using TUG.

#### 2.5.1. Knee Injury and Osteoarthritis Outcome Score

KOOS is a self-reported questionnaire consisting of 42 items in 5 separate subscales of pain, other symptoms, function in daily living (ADL), function in sport and recreation, and knee-related quality of life with acceptable psychometric properties [21,22,23]. Standardized options are given, and each question is assigned a score from 0 to 4. A normalized score (where 100 indicates no symptoms and 0 indicates extreme symptoms) was calculated for each subscale.

#### 2.5.2. Numeric Rating Scale

NRS is a reliable and valid method of measuring pain intensity [24]. Common 11-item NRS was used in which the participant selects a whole number between 0 (no pain) and 10 (worst pain imaginable) that best reflects the intensity of their pain.

#### 2.5.3. Thirty-Second Chair Stand Test

Thirty-second CST is measured as a number of stands where the participants are encouraged to complete as many full stands from a chair as possible within a 30 s time limit. The score is the total number of stands executed correctly within 30 s. This test provides a reasonably reliable and valid indicator of lower body strength in older adults [25,26].

#### 2.5.4. Timed Up and Go Test

TUG measures the time that a person needs to rise from a chair, walk 3 m, turn around, walk back to the chair, and sit down [27]. It has acceptable reliability and has a reasonable predictive value of functional performance following TKA [25,28,29].

### 2.6. Statistical Analyses

Statistical analyses were performed using SPSS 25.0 (IBM, Armonk, NY, USA). The normality of data was checked using the Shapiro–Wilk test, and the homogeneity of variances was checked with Levene’s test. Descriptive statistics were performed for all variables of interest and expressed as median and interquartile ranges.

The distribution of data was not normal except for the variables of body height and body mass index, and we used the Mann–Whitney U test, Fisher’s exact test, and Chi-square test for between-group analyses

For within-group analyses of dependant variables (pre- and post-intervention comparisons), we used Wilcoxon signed-rank test, Fisher’s exact test, and Chi-square test. Results were considered significant for *p* < 0.05. All the analyses included all participants for which data were available.

## 3. Results

**Figure 1:**
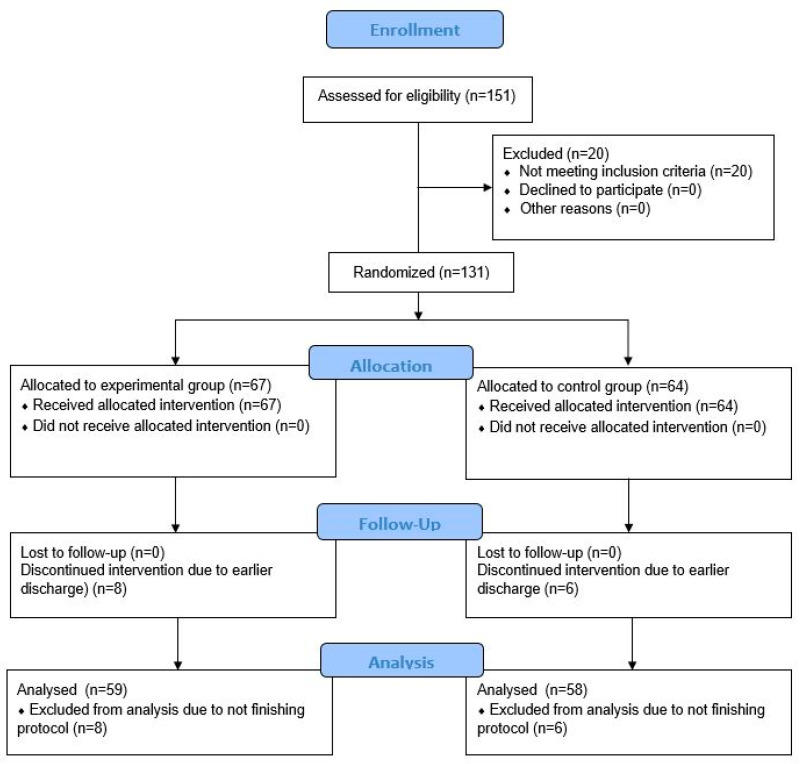
Consolidated Standards of Reporting Trials (CONSORT) flowchart of the study showing recruitment of participants.

A total of 131 participants admitted to inpatient rehabilitation were finally enrolled in the trial and randomized into two groups: 67 to the EG and 64 to the CG. Fourteen participants (10.7%) dropped out of the trial, eight from the EG (11.9%), and six from the CG (9.4%) (Figure 1). Data from 117 participants were included for the final analysis, 59 from the EG and 58 from the CG. The EG and CG were well matched, without differences in the baseline variables (Table 1) (*p* > 0.05). Both groups were comparable in terms of age, sex, body height, body mass, BMI, level of education, operated side of the body, level of the constraint of TKA, surgical technique and approach, place of the surgery, postoperative day, use of walking aid, KOOS, NRS, and functional tests at the beginning of their inpatient rehabilitation. All patients received posterior-stabilized implants using the medial parapatellar surgical approach. The alignment was mechanical in all but six (three from EG and three from CG) patients where kinematic alignment was used. The level of expertise of the surgeons exceeded 50 TKA per year.

Patients in both groups spent 21(0) days hospitalized, and they had 18(0) days of active rehabilitation.

**Table 1:** Baseline characteristics for the experimental and control group.

Variable	EG (N = 59)	CG (N = 58)
Age (years; median (IQR))	70 (10)	69 (9)
Body height (cm; median (IQR))	168 (13)	165 (12)
Body mass (kg; median (IQR))	87 (20)	84 (18)
Body mass index (kg/m^2^; median (IQR))	30.8 (9.3)	30.3 (7.1)
Sex (N (%))		
Male	17 (29)	24 (41)
Female	42 (71)	34 (59)
Education (N (%))		
Secondary level	49 (83)	52 (90)
Tertiary level	10 (17)	6 (10)
Side of the operated knee (N (%))		
Left	25 (42)	32 (55)
Right	34 (58)	26 (45)
Place of the surgery (N (%))		
University hospital	13 (22)	15 (26)
General hospital	46 (78)	43 (74)
Postoperative day at the beginning of the inpatient rehabilitation (day; median (IQR))	64 (84)	70.5 (84)
Use of walking aid upon admission (N (%))		
One crutch	21 (36)	17 (30)
Two crutches	29 (49)	32 (55)
Walker	0 (0)	0 (0)
No use of walking aid	9 (15)	9 (15)
KOOS score (0–100 scale; median (IQR))		
Pain	25 (22)	29.5 (30.8)
Symptoms	32 (32)	32 (33)
ADL function	16 (17)	21.5 (31)
Sport and recreation function	5 (25)	5 (26.3)
Quality of life	44 (31)	38 (34)
NRS (0–10 scale; median (IQR))	2 (5)	3 (5)
30 s chair stand test (no. of stands; median (IQR))	9 (2)	9 (3)
TUG (seconds; median (IQR))	13.8 (5.5)	15 (6.9)

EG—experimental group; CG—control group; IQR—interquartile range; N—Sample size; KOOS—Knee Injury and Osteoarthritis Outcome Score; ADL—activities of daily living; NRS—numeric rating scale; TUG—timed up and go test.

### 3.1. Within-Group Analyses

**Figure 2:**
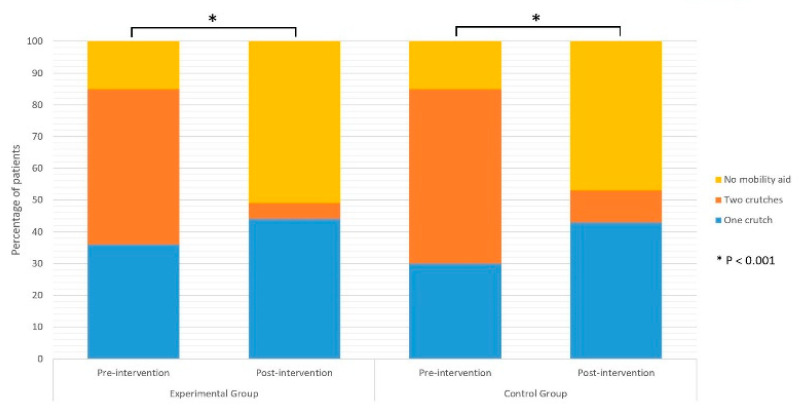
Use of walking aids pre-intervention (day 1) and post-intervention (day 21).

Both groups improved their functional abilities from 1st day of rehabilitation until the 21st day of rehabilitation when final assessments were done. A higher proportion of participants did not use walking aid at the end of rehabilitation in both groups (*p* < 0.05) (Figure 2). In the EG, 85% needed some form of walking aid at the beginning of the rehabilitation, but only 49% of the patients needed walking aid on their 21st day of rehabilitation (*p* < 0.002). Likewise, 85% of the patients from the CG used walking aid on their 1st day of rehabilitation compared to 53% of them on their 21st day of rehabilitation (*p* < 0.001). 

**Figure 3:**
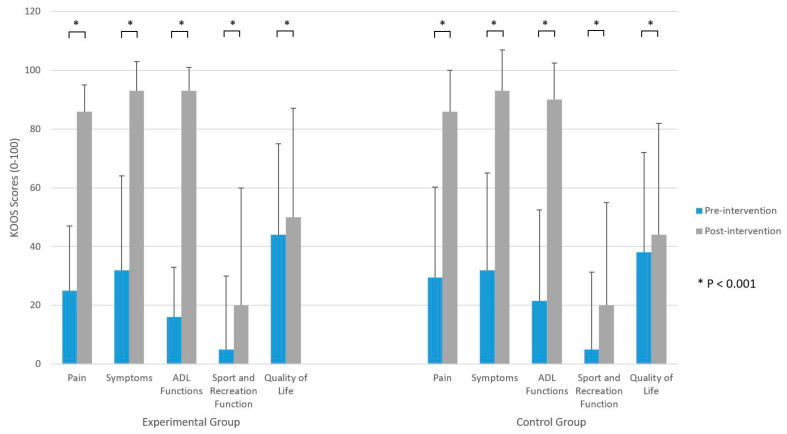
Knee Injury and Osteoarthritis Outcome Score (KOOS) pre-intervention (day 1) and post-intervention (day 21).

KOOS scores improved in both groups when pre-intervention and post-intervention values in all subscales were compared (*p* < 0.001) (Figure 3). Pain subscale improved by 61 points in the EG, and by 56.5 points in the CG. Symptoms score improved by 61 in both groups. ADL function subscale improved by 77 points in the EG, and by 68.5 points in the CG. Sport and recreation function improved by 15 points in both groups. Improvement of the quality-of-life subscale was by 6 points in both groups.

**Figure 4:**
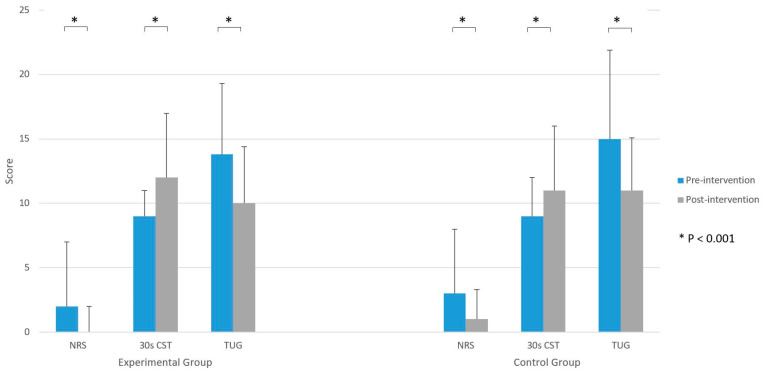
Numeric rating scale (NRS), 30 s chair stand test (CST), and timed up and go test (TUG) scores pre-intervention (day 1) and post-intervention (day 21).

NRS scores lowered significantly (*p* < 0.001), on average, by two points in both groups (Figure 4). Both groups improved their 30 s CST result (*p* < 0.001) (Figure 4). The average difference was the addition of three stands in the EG and two stands in the CG in comparison with the pre-intervention number of stands. The time needed to perform the TUG test was significantly shorter in both groups (*p* < 0.001) (Figure 4). In the EG, the average time period was shorter by 3.8 s, and in the CG, it was shorter by 4 s on average.

### 3.2. Between-Group Analyses

Table 2 shows walking aid use, KOOS and NRS scores, as well as 30 s CST and TUG results on the 21st day of rehabilitation. There were no significant differences between the EG and CG on 21st day of rehabilitation regarding walking aid use (Figure 2). Furthermore, no differences were reported between groups in KOOS and NRS scores at the end of the rehabilitation (Figure 3 and Figure 4). Although the results of KOOS subscales ADL function (*p* = 0.07) and Quality of life (*p* = 0.06) were borderline, there were below the determined level of significance. Additionally, there were no significant differences between groups in their 30 s CST nor TUG test results on their final assessment (Figure 4).

## 4. Discussion

This study aimed to examine the effects of adding EMG-BF to the conventional program of rehabilitation after TKA on the objective and subjective functional outcomes of the patients. The results did not confirm the beneficial effects of adding EMG-BF to the regimen of rehabilitation that consisted of therapeutic exercise performed on land and in the water, education, and electrotherapy, which consisted of interferential current therapy and electrostimulation. There were no significant differences between the EG and the CG regarding pain intensity, patient-reported measure of quality of life, or results of 30 s CST and TUG at the end of the rehabilitation. We were not able to confirm our hypothesis. To the best of our knowledge, only two previous studies have examined the effects of EMG-BF in smaller samples of the TKA population, which makes the comparison of our study with other studies difficult.

EMG-BF is a relatively new method of retraining muscles in orthopedic rehabilitation. It converts myoelectrical signals in the muscle by using surface electrodes which detect a change in muscle activity and feedback this information to the user by visual or auditory signals [17]. Its main use in orthopedic rehabilitation is to increase activity in weak muscles and to improve awareness regarding the contraction and relaxation of the muscle.

One of the previous studies comparing the effects of EMG-BF on a sample of the TKA population was performed by Shanb et al. [30]. They evaluated the effects of adding EMG-BF to active exercise training on m. quadriceps femoris torque, voluntary activation, and functional activity in 45 patients with unilateral TKA. Beneficial effects of adding EMG-BF to the exercise program were found only for The Western Ontario and McMaster Universities Osteoarthritis Index in favor of the EG. The authors did not report significant differences in m. quadriceps femoris torque and voluntary activation between groups. Their sample was younger than ours, with an average age of 60.6 and 60 years for the EG and CG, respectively. Furthermore, Shanb et al. excluded patients with BMI > 30 kg/m^2^, and the BMI of their patients was, on average, 26 and 25 kg/m^2^, respectively. In our study, the median of patients’ BMI was 30.7 kg/m^2^. Additionally, their intervention was different, and it included exercise training sessions performed twice per week for 4 months.

Another previous study, conducted by Wang et al. [31], examined the effects of biofeedback training performed twice daily for 5 days in the early postoperative period after TKA on pain levels. Patients in both groups received continuous passive motion (CPM) therapy; however, those in the EG received 30 min of biofeedback-assisted progressive muscle relaxation during the CPM sessions. Patients in the EG reported less pain caused by CPM than the CG. The study provided support for biofeedback relaxation for pain management in the early postoperative period.

Some other studies investigated the use of EMG-BF on different orthopedic conditions affecting the knee joint. Draper and Ballard [32] reported that EMG-BF is more effective than electrical stimulation in the facilitation of the recovery of peak torque of the quadriceps femoris in patients after anterior cruciate ligament reconstruction. Furthermore, EMG-BF has superior effects regarding m. quadriceps femoris strength and Lysholm Knee Scoring Scale in comparison to home exercise and electrical stimulation after partial meniscectomy [33]. On the other hand, no significant additive effect of EMG-BF on regular strengthening exercise programs in participants with knee osteoarthritis was reported by Yilmaz et al. [34]. Few studies investigated the use of EMG-BF for patellofemoral pain syndrome. Yip and Ng [35], as well as Ng et al. [36], suggested that EMG-BF coupled with exercise provides effective treatment for patellofemoral pain syndrome. On the contrary, Dursun et al. [37] did not find any beneficial effect of adding EMG-BF to the conventional exercise program for patellofemoral pain syndrome.

While the use of EMG-BF seems promising in the rehabilitation of knee disorders, further work is required to justify its use in patients after TKA. Voluntary activation and strength of the m. quadriceps femoris are usually reduced after TKA and m. quadriceps femoris strength in some patients can drop up to 62% of preoperative levels from three to four weeks after surgery [20,38]. This negatively affects the quality of life, mobility, and balance, as well as increases the risk of falling [39]. Conventional strengthening exercise programs used after TKA have limited success in long-term improvements of m. quadriceps function [40], and up to 17% of patients are dissatisfied with the outcome after TKA [41].

Significant efforts to improve patient outcomes after TKA, including implant design, patient optimization, perioperative pain management, and rehabilitation, have been undertaken [12]. Postoperative rehabilitation has a significant contribution to patient outcomes. Despite many rehabilitation modalities available, optimal rehabilitation strategy has yet to be determined, and there is a scarcity of evidence-based practice guidelines and recommendations for postoperative rehabilitation after TKA [12]. Therefore, it is extremely important to investigate the factors that could improve patients’ outcomes after TKA and to identify therapeutic interventions which could be added to the conventional rehabilitation programs to further improve functional outcomes and patient satisfaction scores. Still, their use should be evidence-based, and the cost of providing them should be justified. New equipment, methods, and devices intended to improve patient outcomes arrive on the market and in rehabilitation facilities, but their comparative effectiveness and clinical meaningfulness to the standard of care often remain unknown or uncertain [42].

This study has clinical relevance for patients after TKA. It confirmed that both groups had significant improvements after intensive inpatient rehabilitation. However, the use of EMG-BF did not justify the cost of the device, which could be taken into account when planning to purchase expensive equipment with often uncertain clinical benefits. Expensive equipment does not always give the best results.

The current study has some limitations. First, our period of intervention was only 21 days, which could be possibly too short a period to realize the full potential of EMG-BF on the muscle function. Furthermore, we only did one final assessment on the participants’ final, 21st day of hospital stay. Follow-up after an additional month could have shown some long-term effects of EMG-BF not visible on the 21st day of hospital stay. Additionally, we did not measure m. quadriceps femoris torque and voluntary activation, but we relied only on functional tests to assess the quadriceps strength and function. Quantitative measurements of m. quadriceps femoris torque and voluntary activation would add to the quality of the study. Likewise, our patients started the use of EMG-BF rather late in their rehabilitation, and their BMI was above 30 kg/m^2^ on average. Thus, we cannot generalize our results to the wider population of patients after TKA.

The impact of our research will facilitate further research regarding the use of EMG-BF technology by seeking other modalities of its use that could be more clinically meaningful and efficient. Future research should aim to investigate the long-term effects of EMG-BF in various regimens, including different combinations of biofeedback-assisted isometric and dynamic exercise during longer periods. Additionally, apart from using EMG-BF only on m. quadriceps femoris, it should also be used on the m. gluteus medius since its strength is also diminished in patients after TKA.

In conclusion, data suggest that EMG-BF proves no additional benefits to the rehabilitation after TKA. Conventional rehabilitation consisting of therapeutic exercise, education, and electrotherapy yielded comparable results in terms of patient-reported quality of life, pain intensity, and tests for lower body strength, mobility, and functional performance. Further studies are needed to confirm these findings.

## Figures and Tables

**Table 2:** Walking aid use, KOOS, NRS, 30 s chair stand test and TUG results on 21st day of rehabilitation.

Variable	EG (N = 59)	CG (N = 58)	
Use of walking aid (N (%))			
One crutch	26 (44)	25 (43)	0.557 ^a^
Two crutches	3 (5)	6 (10)	
Walker	0 (0)	0 (0)	
No use of walking aid	30 (51)	27 (47)	
KOOS score (0–100 scale; median (IQR))			
Pain	86 (9)	86 (14)	0.212 ^b^
Symptoms	93 (10)	93 (14)	0.488 ^b^
ADL function	93 (8)	90 (12.5)	0.073 ^b^
Sport and recreation function	20 (40)	20 (35)	0.660 ^b^
Quality of life	50 (37)	44 (38)	0.055 ^b^
NRS (0–10 scale; median (IQR))	0 (2)	1 (2.3)	0.298 ^b^
30 s chair stand test (no. of stands; median (IQR))	12 (5)	11 (5)	0.129 ^b^
TUG (seconds; median (IQR))	10 (4.4)	11 (4.1)	0.143 ^b^

EG—experimental group; CG—control group; IQR—interquartile range; N—Sample size; KOOS—Knee Injury and Osteoarthritis Outcome Score; ADL—activities of daily living; NRS—numeric rating scale; TUG—timed up and go test. ^a^ Chi-Square test; ^b^ Mann–Whitney U test.

## Data Availability

The data presented in this study are available on request from the corresponding author.
